# Inside Alzheimer brain with CLARITY: senile plaques, neurofibrillary tangles and axons in 3-D

**DOI:** 10.1007/s00401-014-1322-y

**Published:** 2014-07-29

**Authors:** Kunie Ando, Quentin Laborde, Adina Lazar, David Godefroy, Ihsen Youssef, Majid Amar, Amy Pooler, Marie-Claude Potier, Benoit Delatour, Charles Duyckaerts

**Affiliations:** 1 Laboratoire de Neuropathologie Escourolle, AP-HP, Hôpital de la Pitié Salpêtrière, 75013 Paris, France; 2 Alzheimer’s and Prion disease team, UPMC Université Paris 06 UMR S 1127, Inserm, U 1127, and CNRS UMR 7225, ICM, 75013 Paris, France; 3 UMR S 968 Inserm/UPMC/CHNO des Quinze-Vingts, Centre de Recherche Institut de la Vision, 75012 Paris, France; 4 Department of Neuroscience, Institute of Psychiatry, King’s College London, DeCrespigny Park, London, SE5 8AF UK

Alzheimer’s disease (AD) is characterized by extracellular deposits of amyloid β (Aβ) peptide and intracellular tau aggregates. Examination of these lesions in *post-mortem* brain tissue routinely provides a two-dimensional picture of the pathology with a limited depth of field. Several tissue-clearing protocols have been recently published [1, 4–6, 8, 10, 11, 16]. We compared the quality of immunostaining on *post-mortem* human brain tissues using several clearing techniques including CLARITY [4, 5], Scale [8], SeeDB [9], and 3DISCO [6]. Results were best with CLARITY. In that method, acrylamide hydrogel links the protein of the tissue and maintains its structure. Removing the lipids makes the block transparent and facilitates antibody diffusion [4, 5]. We noticed that the results were improved if ScaleA2 [8] was used to mount the block after CLARITY.

Formalin-fixed frontal cortex samples from two controls and five AD individuals (Braak VI and Thal 5) were obtained from the Brain Bank GIE NeuroCEB with legal consent. Brain samples were cut at a thickness of 500 μm with a vibratome and were processed by the CLARITY technique [5]. Embedded in an acrylamide hydrogel constituted of 4 % paraformaldehyde (PFA), 4 % acrylamide, 0.25 % temperature-triggering initiator VA-044, PBS, the tissues were passively clarified at 37 °C for 2 weeks in the clearing solution (200 mM boric acid, 4 % w/v SDS, pH 8.5). The blocks were immunostained with a rabbit polyclonal anti-tau B19 antibody [3] and a mouse monoclonal anti-Aβ 4G8 antibody (Covance). Alexa Fluor^®^ 488 goat anti-rabbit and Alexa Fluor^®^ 568 goat anti-mouse antibodies (Life technologies) were used as secondary antibodies. For Aβ and neurofilament double immunohistochemistry, the tissues were first incubated with mouse monoclonal anti-neurofilament antibody (M0762 Dako) and then with Alexa Fluor^®^ 568 goat anti-mouse antibody. After a blocking stage (10 % normal mouse serum), the tissues were incubated with biotin-labelled 4G8 anti-Aβ antibody (Covance), revealed with DyLight 488-labelled streptavidin (KPL, Eurobio, France). Triple staining (Aβ, tau and neurofilament) was also performed: the block was incubated with anti-tau B19 and anti-neurofilament M0762 antibodies revealed with the secondary Alexa Fluor^®^ 488 goat anti-rabbit and Alexa Fluor^®^ 568 goat anti-mouse antibodies. The third antibody was a biotin-labelled anti-Aβ 4G8 antibody (Covance) revealed with streptavidin-Alexa 405 (Life technology). The mounting protocol was modified from the original method: we added a fixation step (4 % PFA in PBS for 15 min) at the end of the procedure to improve the stability of the immunostaining, and an incubation step (0.2 M glycine for 15 min) to quench autofluorescence. We used ScaleA2 solution as mounting medium [8], instead of 80 % glycerol or FocusClear described in the original method [5]. ScaleA2 increased the transparency of the tissue compared to 80 % glycerol, lowered the cost of the experiment compared to FocusClear and shortened incubation time (overnight compared to 2 days). The immunolabellings were analysed with an upright confocal microscope (Olympus Fluoview Fv1000). The Z-stack images were re-constructed using Imaris software (Bitplane).

The volume of the “clarified” block was increased by approximately 40 % after passive removal of the lipids. While 80 % glycerol or FocusClear restored the volume in the original CLARITY technique, ScaleA2 further increased it but kept the morphological quality of the technique. The combination of CLARITY and ScaleA2 is not appropriate for stereological measures because of the volume expansion. Despite the increase in volume, the axons (shown by neurofilament immunostaining) remained continuous (Supplementary 10.1007/s00401-014-1322-y). The clarified tissues were fully transparent. The video, obtained with confocal microscopy, provides a 3-D view of the lesions revealed by Aβ and tau immunohistochemistry (10.1007/s00401-014-1322-y). Amyloid deposits appeared to have diverse 3-D structure in AD brains: some deposits were dense and focal; some diffuse deposits appeared hollow (10.1007/s00401-014-1322-y). Accumulation of Aβ was regularly spaced in clumps throughout the cortex (10.1007/s00401-014-1322-y). Tau accumulated in segmented and discontinuous neuritic processes. Many dystrophic neurites were associated with dense, focal, presumably “mature” Aβ focal deposits [2].

Plaque-induced distorted neurites have been previously reported by in vivo observation in AD transgenic animal [7, 12, 13, 15] and in the affected areas of *post-mortem* human AD brains [10, 14]. Our video of Aβ and neurofilament double immunostaining showed the persistence of a large number of axons, one of them (traced in yellow) deflected by the focal deposits (10.1007/s00401-014-1322-y).

Finally, a triple immunostaining for Aβ (blue), tau (green) and neurofilament (red) showed that the tangle bearing neuron that we identified had no visible axonal projection (10.1007/s00401-014-1322-y). The region of the Aβ focal deposit contained few axons. The Aβ immunoreactivity (revealed with an avidin biotin amplification step) was excellent through the whole thickness of the block. Neurofilament and tau immunoreactivities (revealed with no amplification step) were stronger in a 150-µm-thick layer at the top of the block. The transparency of the block was, however, homogeneous. The 150-µm-thick layer of high immunoreactivity was detected at the top and at the bottom of the block: it was not related to a defect in the penetration of the laser beam during the imaging process but to better antibody penetration near the surface. Longer incubation time, higher concentration of detergent in the buffer  and various amplification systems will be tested in the future to improve the homogeneity of the signal throughout the thickness of the sample.

These first results uncover the 3-D architecture of AD lesions and demonstrate the utility of the CLARITY technique in studying the distribution of AD pathology in 3-D, even in *post-mortem* human tissue.

## Electronic supplementary material

Below is the link to the electronic supplementary material.

**Three-dimensional immunohistological visualization of Aβ and tau lesions in a human post-mortem AD tissue.** A 500-µm-thick tissue of frontal cortex of *post-mortem* human AD brain tissue was clarified and immunostained for Aβ (4G8, *red*) and tau (B19, *green*) for 3 days: primary (1 day) – wash (0.5 day) in PBS-Triton X 0.1 % - secondary (1 day) – wash (0.5 day). The stained tissue was imaged using the 20 × water immersion objective (depth 379 μm, step size = 1 μm) (MP4 23,860 kb)

**Three-dimensional immunohistological visualization of Aβ and neurofilament in a human post-mortem AD tissue.** A 500-µm-thick block of frontal cortex from AD brain was clarified for 7 days and immunostained for Aβ (biotin-labelled 4G8, *green*) and neurofilament (M0762, *red*): primary (1 day) – wash in PBS-Triton X 0.1 % (0.5 day)–secondary (1 day) – wash (0.5 day) – blocking with normal mouse serum (0.5 day) - biotin-labelled antibody (1 day) – wash (0.5 day) – streptavidin-Dylight-488 (1day) – wash (0.5 day) – post-fixation and quenching (1 h) – incubation with ScaleA2 (overnight). The stained tissue was imaged using the 20 × water immersion objective (depth 401 μm, step size = 1 μm). The 3-D image was cropped and neurofilament positive axons were traced and analysed by Imaris filament tracer. The axon that had an increased curvature is shown in yellow and the others in blue (MP4 19,431 kb)

**Three-dimensional immunohistological visualization of Aβ, tau and neurofilament in an AD case.** A 500-µm-thick block of frontal cortex from an AD case was clarified for two weeks and immunostained for tau (B19, *green*), neurofilament (M0762, *red*) and Aβ (biotin-labelled 4G8, *blue*): primary antibodies of rabbit polyclonal B19 anti-tau and mouse monoclonal M0762 anti-neurofilament antibodies (1 day) – wash in PBS-Triton X 0.1 % (0.5 day) – secondary goat anti-rabbit Alexa 488 and goat anti-mouse Alexa 568 antibodies (1 day) – wash (0.5 day) – blocking with normal mouse serum (0.5 day) - biotin-labelled 4G8 antibody (1 day) – wash (0.5 day) – streptavidin-Alexa 405 (1 day) – wash (0.5 day) – post-fixation and quenching (1 h) – incubation with ScaleA2 (overnight). The stained tissue was imaged using the 20 × water immersion objective (depth 418 μm, step size = 1 μm) (MP4 84,678 kb)
